# Regulation of Myogenic Cell Apoptosis, UPS, and Autophagy During Mammalian Skeletal Myogenesis

**DOI:** 10.3390/cells15121061

**Published:** 2026-06-10

**Authors:** Binglin Yue, Wen Hu, Shuo Zhu, Du’an Chen, Huanyu Guan, Zhuoying Zhao, Hui Wang, Jiabo Wang, Jincheng Zhong, Haitao Shi

**Affiliations:** Key Laboratory of Qinghai-Tibetan Plateau Animal Genetic Resource Reservation and Utilization, Sichuan Province and Ministry of Education, Southwest Minzu University, Chengdu 610225, China; yuebinglin123@163.com (B.Y.); 18398573987@163.com (W.H.); 16687104023@163.com (S.Z.); 13982634140@163.com (D.C.); 17208276933@163.com (H.G.); zhao19252026@163.com (Z.Z.); wanghui892321@sina.cn (H.W.); wangjiaboyifeng@163.com (J.W.); zhongjincheng518@126.com (J.Z.)

**Keywords:** apoptosis, UPS, autophagy, skeletal myogenesis

## Abstract

Skeletal myogenesis is an extremely complex process that mononuclear myoblasts undergo proliferation, differentiation, and fusion to form multinucleated contractile muscle fibers, involving a balance between synthesis and degradation metabolism. Skeletal muscle requires an effective mechanism to balance rapid proliferation by degrading supernumerary or damaged organelles/proteins, or by activating cellular signals to regulate subsequent muscle differentiation. In recent years, three important cellular processes—apoptosis, ubiquitin–proteasome system (UPS), and autophagy—have received extensive attention in skeletal myogenesis. The UPS supports the early differentiation process and initiates apoptosis, and the increase in apoptosis activates autophagy to clear damaged organelles and proteins, which in turn inhibits apoptosis, preventing excessive cell death and maintaining cellular stability. The coordination among apoptosis, UPS, and autophagy is more intricate, as they interact through a dynamic balancing mechanism, determining the balance between cell death and survival, and enabling proper muscle differentiation. Here, we explore the molecular signals that mediate apoptosis, UPS, and autophagy, with a focus on analyzing their interrelationship in skeletal myogenesis. Studying the regulatory mechanisms of these molecules will help in understanding the role of cell death in skeletal muscle development, especially how they affect muscle cell differentiation, providing new insights into mammalian skeletal myogenesis.

## 1. Introduction

From a metabolic perspective, the regulation of skeletal myogenesis depends on the coordinated balance between anabolism and catabolism. During skeletal myogenesis, cells need to remove damaged or unnecessary proteins and even cells in a timely manner to balance excessive anabolism and maintain tissue homeostasis [1]. According to the specific environment, stimuli, and developmental state of the cells, mammalian skeletal muscle is primarily induced to undergo cell death through apoptosis, autophagy, and necrosis [2,3]. Apoptosis is the first identified form of programmed cell death, involving the activation of metabolic enzymes, particularly proteases, in response to signals resulting from an extrinsic pathway (receptor-mediated pathway) or an intrinsic pathway (the mitochondrial pathway). This leads to the rapid destruction of cellular structures and organelles, accompanied by DNA fragmentation, nuclear condensation, and the formation of apoptotic bodies, which are then phagocytosed by macrophages or neighboring cells without triggering an inflammatory response [4,5]. In highly proliferative myoblasts, apoptosis helps eliminate excess or metabolically redundant cells to prevent over-proliferation and maintain a stable cell number. However, in mature multinucleated myofibers, apoptosis exhibits some unique characteristics: unlike other mononucleated cells, the activation of the apoptotic cascade leads to the removal of only a single myonucleus and its corresponding cytoplasmic portion, resulting in muscle fiber atrophy rather than complete cell death [6]. Autophagy is another important mechanism for regulating muscle mass under catabolic conditions, capable of rapidly transporting cytoplasmic components to lysosomes for degradation and recycling [7,8]. Additionally, autophagy can promote muscle differentiation and regeneration by modulating complex cellular signaling pathways [9]. Although autophagy is generally considered a protective mechanism for cells, excessive activation of autophagy in certain circumstances can also lead to muscle atrophy and even result in the displacement of myonuclei, ultimately causing muscle cell death [10]. In contrast to apoptosis and autophagy, necrosis is often regarded as a passive, non-programmed form of cell death, typically resulting from severe cellular damage (e.g., ischemic or toxic injury) and not regulated by specific mechanisms, which is characterized by strong inflammatory responses and fragmentation of the sarcoplasm [11,12,13]. Moreover, the UPS is one of the primary cellular pathways that maintain homeostatic protein levels, and it is composed of the ubiquitin-conjugating system and the 26S proteasome, the latter serving as its proteolytic core. Previous studies have shown that the UPS ensures precise regulation of cell-cycle transitions and mitotic entry, and it regulates myogenesis by targeting muscle-specific proteins [14,15,16].

It has been recognized that different types of cell death overlap. For example, the molecule Beclin-1, which was previously defined as an apoptosis-activating mediator, also acts as a mediator for autophagy activation, indicating the existence of crosstalk between apoptosis and autophagy [9,17]. Receptor- and mitochondria-mediated apoptosis can first induce autophagy to prevent further cell death, while abnormal autophagic activity may trigger apoptosis through mitochondrial pathways [18,19]. There is a complex interplay between the UPS and autophagy, where core components of one system often become degradation targets of the other, and this cross-regulatory mechanism ensures precise control of intracellular protein homeostasis [20,21]. In addition, as a highly target-specific system for protein degradation, the UPS can also tightly regulate apoptotic proteins through various E3 ligases to either enhance or suppress apoptosis [22,23]. However, the specific details of their relationship remain unclear. In this review, we focus on the relationship among apoptosis, UPS, and autophagy during mammalian skeletal myogenesis, as well as their coordinated roles in muscle differentiation, particularly involving mitochondria as central regulators of both processes. Understanding these mechanisms will deepen our comprehension of mammalian skeletal myogenesis.

## 2. Apoptosis

Apoptosis is a highly conserved programmed cell-death mechanism: cells undergo orderly and precise self-destruction in response to an external or internal pathway, thereby preventing potential damage or disease. The typical features of apoptosis include the cell undergoing cytoplasmic condensation, the formation of blebbing structures on the cell membrane, chromatin condensation, and DNA fragmentation. Among these, the most distinctive feature is the externalization of phosphatidylserine (PS) on the surface of the apoptotic cell membrane, which serves as a recognition site for phagocytosis [24]. Importantly, the integrity of the cell membrane is maintained until the late stages of apoptosis, preventing the activation of inflammatory responses. In addition, the loss of mitochondrial membrane potential leads to increased membrane permeability, which is an early hallmark of apoptosis [25].

**Figure 1:**
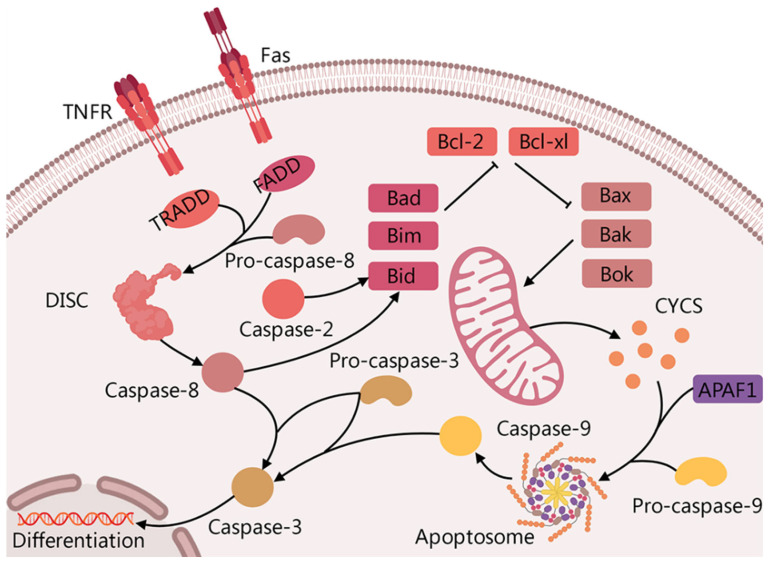
Schematic representation of the extrinsic and intrinsic apoptotic signaling pathways. Apoptosis is mediated through two main pathways. The extrinsic pathway: The binding of death ligands (e.g., TNF or FasL) to surface receptors recruits adaptor proteins (TRADD/FADD) and procaspase-8 to form the death-inducing signaling complex (DISC). Activated caspase-8 directly initiates the effector caspase cascade or cleaves Bid into tBid to engage the mitochondrial pathway. The intrinsic (mitochondrial) pathway: Stress signals activate BH3-only proteins, which neutralize anti-apoptotic Bcl-2 proteins and activate Bax/Bak. This induces mitochondrial outer membrane permeability (MOMP), releasing cytochrome c (CYCS) and AIF. CYCS facilitates the assembly of the apoptosome (with APAF1 and caspase-9), leading to the activation of executioner caspase-3 and subsequent cell death.

Generally, the extrinsic pathway occurs through ligand-mediated death receptor signaling: ligands (e.g., TNF or FasL) bind to their corresponding death receptors, inducing a series of cysteine-dependent, aspartate-specific protease (caspase) signaling cascades to amplify the apoptotic signal [26,27]. Caspases typically exist in an inactive form in the cytoplasm, known as procaspases, which can be activated through dimerization or proteolytic cleavage, becoming enzymatically active caspases [28]. In the active caspase-signaling cascades, initiator caspases (e.g., caspase-8, caspase-9, and caspase-12) can further activate effector caspases (e.g., caspase-3 and caspase-6), which then execute proteolytic events that lead to apoptosis [29,30]. For instance, the binding of TNF/FasL to its receptor TNFR1/Fas lead to the recruitment of the adaptor protein TNF receptor-associated protein with death domain (TRADD)/Fas-associated protein with death domain (FADD), which bring procaspase-8 to the plasma membrane, forming the death-inducing signaling complex (DISC) to activate caspase-8 [31,32]. In fact, the active caspase-8 released into the cytoplasm can generate a dual apoptotic signal: on the one hand, caspase-8 directly processes and activates caspase-3, leading to the cleavage of key structural proteins of the plasma membrane and nuclear envelope, as well as the activation of DNA endonucleases [33,34]. On the other hand, caspase-8 cleaves the BH3-only protein Bid, generating the truncated carboxy-terminal fragment tBid, which further promotes the permeability of the mitochondrial outer membrane (MOM), leading to the release of cytochrome c (CYCS) and the activation of the mitochondrial pathway [35,36] (Figure 1).

The mitochondrial pathway is an intrinsic apoptotic pathway that involves changes in mitochondrial membrane permeability regulated by Bcl-2 family proteins, leading to the release of pro-apoptotic factors such as CYCS, which in turn activate the caspase cascade to initiate apoptosis [37,38]. Based on the conservation of Bcl-2 homology (BH) domains, Bcl-2 family proteins can be classified into anti-apoptotic proteins (e.g., Bcl-2, Bcl-xL, and Mcl-1), pro-apoptotic proteins (e.g., Bax, Bak, and Bok), and BH3-only proteins (e.g., Bad, Bid, and Bim). Under normal conditions, anti-apoptotic proteins maintain mitochondrial membrane stability by binding to and inhibiting the activity of pro-apoptotic proteins. However, when stress signals within the cell trigger the activation of BH3-only proteins, their interactions with both anti-apoptotic and pro-apoptotic proteins ultimately activate Bax and Bak to induce mitochondrial membrane permeability [39,40,41,42,43]. Mitochondrial membrane permeability is a key determinant of whether a cell undergoes intrinsic apoptosis: the mitochondrial outer membrane permeability (MOMP) leads to the leakage of CYCS into the cytoplasm, where it binds to apoptotic protease-activating factor 1 (APAF1) and procaspase-9 to form the apoptosome, a heptameric structure, ultimately leading to the cleavage and activation of caspase-3 [44] (Figure 1). In addition, the MOMP can also lead to the release of nuclease G and apoptosis-inducing factor (AIF), which induce apoptosis in a caspase-independent manner [45,46].

Current studies suggest that there are three distinct mitochondrial channels that mediate mitochondrial membrane permeability: the mitochondrial apoptosis-inducing channel (MAC) and the voltage-dependent anion channel (VDAC) on the MOM, as well as the mitochondrial permeability transition pore (MPTP) on the inner membrane [47]. The MAC, precisely regulated by Bcl-2 family proteins, is the primary mechanism leading to cell apoptosis. In healthy cells, Bax and Bak do not maintain a stable membrane-bound state for long periods but instead continuously shuttle between the cytosol and the MOM, maintaining a dynamic equilibrium [48,49]. Once subjected to stress, activated Bax and/or Bak oligomerize on the MOM to form the MAC [50,51]. Unlike the voltage-insensitive MAC, VDAC is the most abundant hydrophilic protein channel on the MOM, involved in the transport of Ca^2+^ and metabolites and in energy production, as well as the structural and functional interactions between the mitochondria and the endoplasmic reticulum (ER) [52,53]. Three different isoforms of VDAC (VDAC1, VDAC2, and VDAC3) have been identified in mammals, and VDAC1 and VDAC2 are the most extensively studied isoform. In response to pro-apoptotic signals, VDAC1 undergoes oligomerization and forms larger pores on the MOM, allowing for the release of mitochondrial pro-apoptotic proteins [54]. In addition, VDAC1 also regulates apoptosis through its direct interactions with the anti-apoptotic proteins Bcl-2 and Bcl-xL [55,56]. It has been suggested that interaction between VDAC2 and Bax promotes Bax-mediated apoptosis, which is markedly different from BAK: VDAC2 limits apoptosis by sequestering Bak, while specific BH3-only proteins can restore Bak activity and trigger apoptosis through their interactions with VDAC2 [57,58].

mPTP is a non-selective channel that allows for the passage of solutes smaller than 1.5 kDa. The sustained opening of mPTP can lead to matrix swelling, followed by MOM rupture, and the non-specific release of intermembrane space proteins into the cytosol, which is typically associated with late-stage apoptosis and necrosis [59]. Actually, the activation of mPTP may be a consequence of apoptosis, rather than its cause. The active mPTP does not directly initiate the apoptotic process, but is instead triggered by various intracellular stress signals, such as calcium overload, oxidative stress, and ATP depletion. The sustained opening of mPTP further disrupts mitochondrial function by inducing mitochondrial swelling and membrane damages and leads to a more complete release of apoptotic factors, thereby ensuring the full execution of the apoptotic program. But so far, the molecular composition of mPTP has not been fully clarified [47]. The latest proposals suggest that the mitochondrial F_1_F_0_ (F)-ATP synthase is key component of the mPTP, and adenine nucleotide translocator (ANT), matrix cyclophilin D (CypD), phosphate carrier (PiC), and the translocator protein (TSPO) are only modulators of the mPTP complex [60]. Previous studies have shown the MAC and VDAC all contribute to the mPTP channel; however, knockout experiments indicate that their roles in the formation of the mPTP complex are dispensable [61]. In intrinsic apoptosis, these channels may act individually or in combination to ensure the execution and amplification of apoptotic signals. There is also an intrinsic apoptosis pathway independent of the mitochondria, involving caspase-12, a member of the caspase family located on the ER membrane. Unlike the release of CYCS from the mitochondria to activate caspase-9 and caspase-3, disruptions in intracellular calcium homeostasis can induce the activation of caspase-12 through calpain, a calcium-dependent protease. This, in turn, triggers downstream caspase-9 and caspase-3, either directly or indirectly, leading to cell apoptosis [62]. Moreover, activated calpain can also cleave Bcl-xl, causing the loss of its anti-apoptotic activity and thereby trigger the activation of downstream pro-apoptotic signals [63].

## 3. UPS and Autophagy

As mentioned above, different signaling pathways regulate the process of cell apoptosis. However, the nuclear fragments, damaged organelles, and unfolded proteins generated during apoptosis still require efficient clearance systems for removal. The UPS and autophagy are two major intracellular degradation mechanisms responsible for this process; they can function independently or cooperate with each other and are all closely associated with ubiquitination.

**Figure 2:**
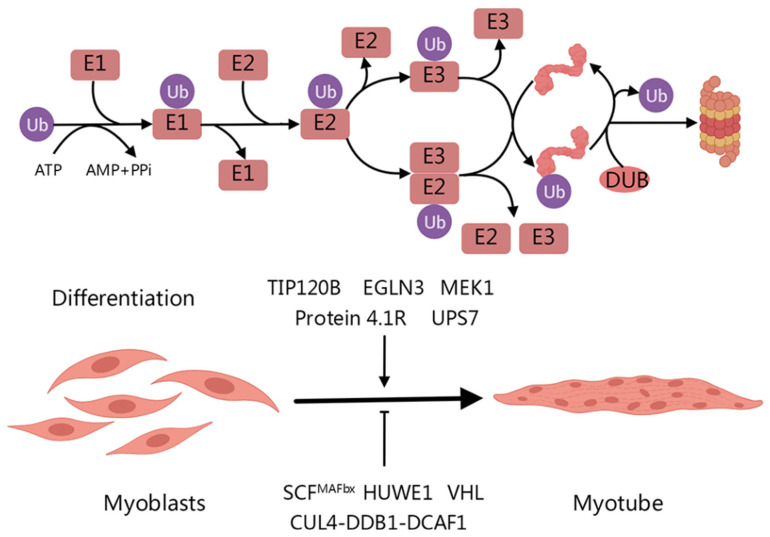
The ubiquitin–proteasome system (UPS) orchestrates myogenesis through cell cycle control and protein turnover. Ubiquitination is a sequential process catalyzed by three key enzymes—ubiquitin-activating enzyme (E1), ubiquitin-conjugating enzyme (E2), and ubiquitin ligase (E3)—which coordinate to covalently attach ubiquitin to specific substrates. The UPS acts as a precise regulator of myogenic differentiation via two distinct mechanisms. Cell cycle regulation: the SCF complex regulates the G1-to-S transition and mitotic entry by targeting key cell cycle regulators (e.g., cyclin E, p21, and p27). Regulation of myogenic factors: the stability of key transcription factors is determined by the balance between E3 ligases and stabilizing factors. MyoD is targeted for degradation by SCF^MAFbx^, HUWE1, and CUL4 (promoted by G9a methylation), whereas MEK1-mediated phosphorylation stabilizes it. Similarly, Myogenin (Myog) turnover is mediated by SCF and VHL ligases but is antagonized by TIP120B, EGLN3, and the deubiquitinase USP7.

Ubiquitination is a series of complex enzyme-catalyzed reactions. A highly conserved 76-amino-acid protein, ubiquitin is covalently attached to a target protein via ubiquitin chains, under the coordinated action of ubiquitin-activating enzyme (E1), ubiquitin-conjugating enzyme (E2), and ubiquitin ligase (E3) [64,65] (Figure 2). Ubiquitin molecules can attach to substrate proteins in different ways, including variations in the number of ubiquitin molecules (monoubiquitination, multi-monoubiquitination, and polyubiquitination) [66]. In a polyubiquitin chain, the seven lysine (Lys) residues of ubiquitin (Lys6, Lys11, Lys27, Lys29, Lys33, Lys48, and Lys63), as well as its N-terminal methionine (Met1), can form covalent bonds with other ubiquitin molecules, thereby generating homotypic or heterotypic polyubiquitin chains [67,68]. In addition, ubiquitin itself can undergo post-translational modifications, such as phosphorylation, acetylation, and SUMOylation, thereby expanding the functional scope of ubiquitination in the cell [69].

The key to the UPS is the recognition of polyubiquitin chains by the 26S proteasome, which translocates the target proteins into its proteolytic core, where ATP-dependent proteolysis occurs. Polyubiquitin chains can be recognized by the intrinsic ubiquitin receptors of the proteasome (e.g., Rpn1/PSMD2, Rpn10/PSMD4, or Rpn13/ADRM1) or by shuttle factors containing ubiquitin-binding domains (UBDs) and proteasome-binding domains (PBDs) (e.g., Ubl or PB1), which facilitate translocation to the proteasome [70]. Lys48-linked polyubiquitination and monoubiquitination (especially proteins smaller than 150 amino acids) are widely recognized as the hallmark that induces target proteins for proteasomal degradation. Additionally, heterotypic polyubiquitin chains, such as Lys11/Lys48, Lys29/Lys48, and Lys48/Lys63, have also been suggested to facilitate proteasomal degradation, highlighting that the structure and type of ubiquitin chains are critical for initiating the UPS. Prior to target proteins’ degradation, the polyubiquitin chains are removed by the deubiquitinating enzymes (DUBs) of the proteasome, allowing the ubiquitin molecules to be recycled for reuse [71,72,73]. An increasing number of studies suggest that the UPS typically handles small, single polypeptides or proteins, while larger or more structurally complex substrates, such as macromolecular complexes (e.g., the protein, glycogen, lipid, and nucleotide) and organelles (e.g., the mitochondria, peroxisome, and ER), cannot pass through the proteasomal channel directly and, thus, require degradation via the autophagy [74,75].

**Figure 3:**
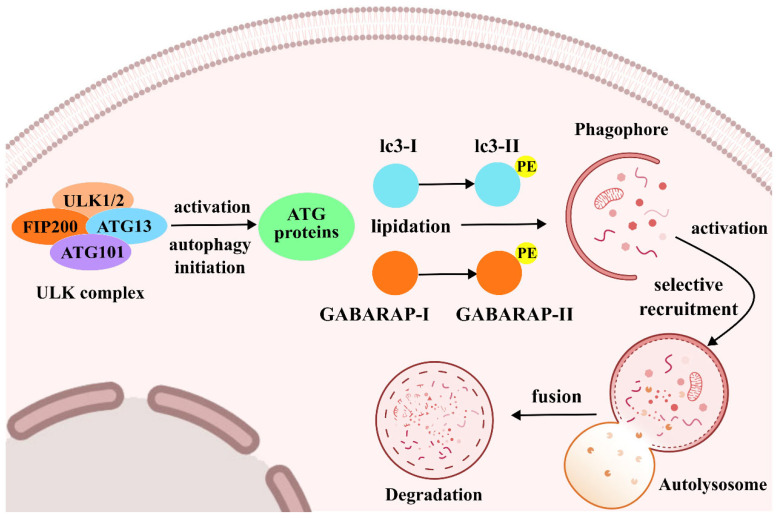
The molecular machinery of macroautophagy. The process initiates with the activation of the ULK complex (ULK1/2, ATG13, FIP200, and ATG101). ATG proteins facilitate the conversion of LC3 and GABARAP from cytosolic forms (I) to lipidated forms (II), which conjugate to phosphatidylethanolamine (PE) to drive phagophore expansion. Specific substrates are recruited via autophagy receptors containing LIR or GIM motifs that bind to lipidated LC3/GABARAP, ensuring selective encapsulation and subsequent lysosomal degradation.

Autophagy is a conserved lysosomal degradation pathway, which can be classified into three types based on the different mechanisms of substrate delivery to the lysosome: macroautophagy, microautophagy, and chaperone-mediated autophagy (CMA) [76,77]. In macroautophagy, substrates are encapsulated within autophagosomes and subsequently fuse with lysosomes to form autolysosomes; microautophagy does not involve the autophagosome formation; instead, the lysosomal membrane invaginates to directly engulf substrates into the lysosome; for CMA, substrates are selectively recognized by molecular chaperones (e.g., p62) and directed into the lysosome for degradation [78,79]. Macroautophagy is the most typical form of autophagy (hereafter referred to as autophagy), which is characterized by the initial formation of a specific membrane structure, phagophore, and subsequently expands to form the double-membrane autophagosome. The process is facilitated by abundant autophagy-related proteins (ATGs), for example, the activation of the ULK complex (composed of ULK1/2, ATG13, FIP200, and ATG101) promotes the transformation of ATG8 family members microtubule-associated protein 1 light chain 3 (LC3) and γ-aminobutyric acid receptor-associated protein (GABARAP), from unlipidated forms (LC3-I and GABARAP-I) to lipidated forms (LC3-II and GABARAP-II). By binding to phosphatidylethanolamine (PE) through their terminal fatty acid chains, these lipidated forms are accumulated on the autophagosomal membrane and facilitate the membrane expansion and closure, serving as the important markers of autophagic activity (Figure 3). In this process, specific substrates are selectively delivered to LC3/GABARAP-marked autophagosomes through autophagy receptors containing an LC3-interacting region (LIR) or GABARAP-interacting motif (GIM). These receptors bind to LC3 or GABARAP proteins through their LIR or GIM sequences, ensuring substrates are accurately recognized and delivered to the autophagosomes, where they are subsequently involved in lysosomal fusion and degradation [80].

In addition, these autophagy receptors often contain UBDs as well, which connect substrates to the autophagosomal membrane in a ubiquitin-dependent manner. In fact, all stages of autophagy are finely regulated by ubiquitination: autophagy-related proteins, such as the ULK1 complex, beclin-1, LC3, and endosomal sorting complex required for transport (ESCRT), are themselves ubiquitinated, which affects their function and their interactions with other complexes [81,82]. Research has shown that Lys63-linked polyubiquitin chains are preferentially directed to the lysosome over Lys48-linked polyubiquitin chains, through autophagy receptors such as p62. Actually, p62 can act as both a selective autophagy receptor (via its LIR) and a proteasomal shuttling factor (via its PBD), with the pathway selection depending on the oligomeric state of p62. On the one hand, p62 dimers recognize individual protein molecules tagged with Lys48-linked polyubiquitin chains through its UBD and transport the ubiquitinated proteins to the proteasome via its PBD. On the other hand, binding to Lys63-linked polyubiquitin chains, a high cellular concentration of free ubiquitin and even the N-degradation products bound by the ZZ domain of p62 independently of Ub can induce p62 oligomerization, which facilitates the autophagic pathway through LIR-mediated interactions [72,83].

It is becoming increasingly clear that autophagy also participates in the lysosomal degradation of non-protein cellular components, such as mitochondria (mitophagy), ER (ER-phagy), ribosome (ribophagy), and lipid (lipophagy), involving ubiquitin chain types such as Lys63 (and likely others) [84,85]. For example, during mitophagy, the E3 Parkin is recruited to damaged mitochondria and mediates the ubiquitination of mitochondrial proteins through Lys63 and Lys6/Lys11 linkages, generating mitophagy signals [86,87]. Additionally, ubiquitin-independent autophagy receptors (such as BNIP3, NIX/BNIP3L, FUNDC1, and BCL2L13) can also anchor to mitochondria through their transmembrane domains, activating autophagy signals [88]. Interestingly, there is a UBL domain within Parkin that can facilitate its translocation to the proteasome. Disruption of Parkin’s interaction with the proteasome leads to a significant delay in mitochondrial clearance, suggesting that the proteasome may be involved in the early stages of mitophagy by clearing ubiquitin-tagged damaged mitochondria [89,90].

An increasing number of studies show that the UPS and autophagy pathways cooperate and are subject to complex feedback regulation, and these processes are associated with the unfolded protein response (UPR), p53, and the N-terminal arginylation pathway. For example, inhibition of the UPS (using agents such as bortezomib or MG132) can result in the accumulation of unfolded or misfolded proteins in the ER lumen. This accumulation triggers the dissociation of ER membrane receptors, including protein kinase-like endoplasmic reticulum kinase (PERK), inositol-requiring enzyme 1 (IRE1), and activating transcription factor 6 (ATF6), subsequently activating the UPR signaling pathway. Specifically, PERK activation phosphorylates eIF2α, enhancing the synthesis of the transcription factor ATF4, which subsequently promotes the expression of ATG5, ATG7, and LC3, thereby initiating autophagy [91,92]. By sensing ER stress signals, IRE1 activates its downstream c-Jun N-terminal kinase (JNK) pathway to promote core autophagy genes expression. Meanwhile, JNK can phosphorylate Bcl-2, relieving its inhibition of Beclin-1, thereby promoting the formation of autophagosomes [93,94]. Studies have shown that ATF6, which translocates from ER to the nucleus, can form a heterodimeric transcription factor with C/EBP-β, inducing the expression of death-associated protein kinase 1 (DAPK1). DAPK1 then promotes the maturation of autophagosomes by phosphorylating Beclin-1 [95]. Moreover, under ER stress, nuclear factor erythroid 2-related factor 2 (NRF2) is released from the Kelch-like ECH-associated protein 1 (KEAP1) complex and translocates into the nucleus, where it indirectly promotes autophagy by upregulating ATGs [96,97].

Under UPS inhibition, the nuclear accumulation of the transcription factor p53 promotes autophagy by inducing the expression of autophagy-related genes, such as the damage-regulated autophagy modulator (p21), while cytoplasmic p53 promotes autophagy through activation of AMPK or inhibits autophagy gene expression by suppressing the nuclear translocation of transcription factor TFEB [98,99]. This indicates that p53 plays a dual role in the cell’s response to stress and in maintaining cellular homeostasis. Vice versa, autophagy inhibition enhances UPS by increasing the expression of the proteasome catalytic subunit β5 (PSMB5), and it can also promote UPS by upregulating ROS to interfere with the redox potential of FoxO [100,101]. Additionally, since the proteasome has been identified as a target for autophagic degradation (proteasome autophagy), the increased proteasome peptidase activity following autophagy inhibition may be associated with the accumulation of the proteasome. However, inhibition of autophagy leads to excessive accumulation of ubiquitinated proteins and p62 aggregates, which isolating ubiquitinated proteins from the proteasome or inactivating UPS regulators (such as p97/VCP) to delay proteasomal degradation of proteins, and this perspective has been further validated in Atg7/Atg5-deficient mice [102,103]. Furthermore, due to defects in proteasome autophagy, leading to the accumulation of aged or damaged proteasomes. This, in turn, results in proteasome system overload and dysfunction. Thus, it is evident that complex interplay between UPS and autophagy exists, where core components of one system often become degradation targets of the other. The proteasome, in addition to being a selective target of autophagic degradation, can degrade ULK1 to regulate autophagy activity, ensuring the timely initiation and termination of autophagy through a feedback mechanism [21]. Furthermore, ATG proteins can be ubiquitinated and degraded via the UPS, while deubiquitinases may prevent Beclin-1 from being degraded by the proteasome by removing its ubiquitin chains, thereby maintaining the dynamic balance of autophagy [20]. All in all, this cross-regulatory mechanism ensures precise control of intracellular protein homeostasis.

## 4. Connection Among the Apoptosis, UPS, and Autophagy in Skeletal Myogenesis

**Figure 4:**
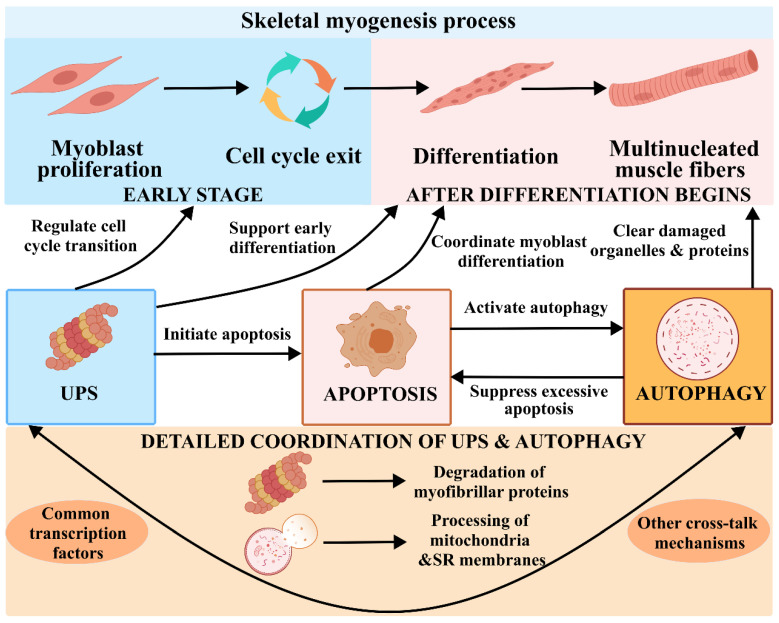
Cross-regulation mechanisms of degradation systems (UPS, apoptosis, and autophagy) during skeletal muscle differentiation. During the multi-step process of skeletal myogenesis, three major degradation systems synergistically drive cellular and organelle remodeling through complex crosstalk mechanisms. Early differentiation: The UPS supports the differentiation process by precisely regulating cell cycle transitions and initiating apoptosis. Differentiation initiation and feedback regulation: increased levels of apoptosis activate autophagy to clear damaged proteins and organelles; meanwhile, autophagy maintains cellular stability by suppressing excessive apoptosis. System synergy and remodeling: the UPS (which primarily mediates the degradation of myofibrillar proteins) and the autophagy–lysosomal pathway (which primarily processes organelles such as mitochondria and the sarcoplasmic reticulum) maintain a dynamic balance, potentially regulated by shared transcription factors. The rapid induction and synergistic action of these degradation pathways efficiently eliminate pre-existing cellular structures and debris, ultimately driving myoblast differentiation and tissue remodeling.

Skeletal myogenesis is a complex multi-step process involving myoblast proliferation, cell cycle exit, and subsequent differentiation and fusion into multinucleated muscle fibers. Especially, skeletal muscle differentiation is accompanied by organelle remodeling and significant morphological changes, and growing evidences indicate that various degradation systems such as the apoptosis, UPS, and autophagy critically relevant to myoblast differentiation. These three systems regulate skeletal muscle differentiation in a complex cross-regulation mechanism: During the early stages of differentiation, the UPS supports the differentiation process by regulating the precise transition of various cell cycle phases and initiating apoptosis. Once differentiation begins, the increased level of apoptosis activates autophagy to clear damaged organelles and proteins. In turn, autophagy suppresses excessive apoptosis, maintaining cellular stability. Furthermore, the coordination between UPS and autophagy is more intricate, as they interact through a dynamic balancing mechanism, ensuring proper skeletal muscle differentiation (Figure 4).

### 4.1. Apoptosis in Skeletal Myogenesis

The apoptosis of myoblasts is tightly regulated by initiator and effector caspases. Activation of initiator caspases (such as caspase-2, -8, and -9) marks the onset of apoptotic signaling, amplifying the process through a signaling cascade. Subsequently, effector caspases (such as caspases-3) are activated, executing the destruction of cellular structures and functions, ultimately leading to cell death. The molecular pathways through which caspases regulate apoptosis have been well elucidated; however, their non-apoptotic functions are not fully understood. For example, caspase-3 is rapidly and transiently activated during myoblast differentiation, coinciding with the expression of classical myogenic markers. This process is inhibited by reduced caspase-8 and caspase-9 expression or overexpression of the anti-apoptotic protein Bcl-xl, revealing the non-canonical roles of receptor-mediated and mitochondrial apoptotic pathway in supporting myogenic differentiation and fusion without triggering cell death [104,105,106]. The coordination between apoptosis and myogenic differentiation appears to be mediated predominantly through the intrinsic (mitochondrial) pathway. Pharmacological or genetic inhibition of mitochondrial apoptotic components (e.g., APAF1 inhibitor M50054, siRNA against CYCS or APAF1, or overexpression of Bcl-2/Bcl-xL) significantly impairs myotube formation and fusion [105]. Although components of the extrinsic pathway (e.g., reduced caspase-8) can also affect differentiation, the majority of evidence highlights MOMP, cytochrome c release, apoptosome assembly, and downstream caspase-9/caspase-3 activation as the critical non-apoptotic signals supporting myoblast differentiation. Extrinsic pathway components may provide auxiliary input, but intrinsic mitochondrial signaling constitutes the dominant route linking apoptosis-related machinery to successful myogenesis. Collectively, these studies demonstrate that disruption of the mitochondrial apoptosis signaling pathway impedes the myogenesis process. In addition, it has been reported that cultured myoblasts exhibit minimal changes in cytosolic and nuclear AIFM1 levels during differentiation, and current evidence suggests that mitochondria-derived nucleases, such as AIFM1 and ENDOG, appear to play little or no significant role in myogenic differentiation [107].

The potential mechanism by which the caspase-3 activation promotes muscle differentiation may be associated with its cleavage of specific substrates involved in the myogenic program. Fernando et al. identified mammalian sterile twenty-like kinase 1 (MST1) as a substrate of caspase-3. Caspase-3-dependent activation of MST1 helps enhance downstream members of the MAPK cascade (MKK6 and p38 MAPK), thereby effectively promoting myoblast differentiation [108]. Larsen’s study suggests that caspase-activated DNase (CAD) is involved in the formation of transient genome-wide DNA damage/strand breaks during the early stages of skeletal muscle differentiation. Caspase-3-mediated CAD induces strand breaks in the promoter region of the key cell cycle regulator p21, activating p21 gene expression to drive myoblast differentiation [33]. Moreover, caspase-2 acts upstream of caspase-3 activation, exerting a more profound regulatory control over the differentiation process, though the specific mechanism remains controversial [109]. This includes cytoplasmic caspase-2 cleaving Bid into tBid, leading to mitochondrial CYCS release and subsequent activation of the mitochondrial apoptotic pathway; nuclear caspase-2 induces myogenic differentiation by regulating various aspects of cell cycle exit through p21; caspase-2 may directly activate caspase-3 by interacting with its prodomain [105,110,111,112]. Interestingly, this study revealed that in cells treated with siCaspase-2 and siBid, myosin expression was suppressed by 80% and myoblast fusion was inhibited by 90%, whereas siAPAF1- or siCYCS-treated myoblasts exhibited a lesser degree of inhibition (both ~40%) [105]. These data demonstrate that upstream suppression of mitochondrial apoptosis signaling via siCaspase-2 and siBid exerted a more pronounced inhibitory effect on differentiation compared to the lack of downstream signaling caused by blocking apoptosome formation.

When differentiation is induced in culture, typically by serum starvation or low-mitogen conditions, approximately 30% of myoblasts undergo apoptosis rather than differentiation [113,114]. A substantial portion of this apoptosis is directly attributable to the abrupt withdrawal of growth factors and nutrients inherent to standard in vitro differentiation protocols. In contrast, in vivo myogenesis and muscle regeneration occur in a nutrient-rich physiological environment without systemic starvation. Therefore, while in vitro models effectively recapitulate key molecular events linking apoptosis and differentiation, the extent and triggers of apoptosis observed in vitro are amplified by these artificial conditions and should be interpreted cautiously when extrapolating to physiological contexts. MyoD is a classic bHLH (basic helix-loop-helix) transcription factor that regulates differentiation by inducing muscle-specific genes during the early stage of myogenesis [115,116]. Upstream, the cyclin–Cdk complex inhibits MyoD activity through phosphorylation, while cell cycle inhibitors can relieve this suppression. For example, ectopic expression of p16, p21, and p57 inhibits cyclin–Cdk-dependent MyoD phosphorylation, triggering myoblast differentiation [117,118]. Evidence also suggests that p57 can directly interact with MyoD to induce muscle-specific genes [119]. Additionally, ectopic MyoD expression can activate p21, p57, and pRb through distinct pathways, inducing cell cycle exit to facilitate myoblast differentiation [120,121,122]. Interestingly, some studies show that silencing MyoD also impairs myoblast apoptosis, which disrupting MyoD’s direct binding to the promoter of the pro-apoptotic Bcl-2 family member PUMA [123,124,125]. Thus, MyoD can serve as a critical branching point for myoblasts exiting the cell cycle, determining whether the cells undergo apoptosis or initiate differentiation.

Some studies have hypothesized that cellular differentiation may represent a attenuated form of apoptosis, with conserved biochemical pathways of myoblast apoptosis serving as integral components of their terminal differentiation [126]. For example, caspase activity during differentiation is transient and has been reported to be of lower intensity compared to that observed during apoptosis [107]. This delicate modulation of apoptotic signaling may enable surviving cells to evade death while progressing through the myogenic program. Furthermore, APAF1 levels progressively decline during myoblast differentiation, suggesting reduced apoptosome formation, whereas excessive activation of mitochondrial apoptosis signaling leads to excessive degradation of cellular components and DNA fragmentation, ultimately resulting in myoblast death and diminished differentiation [107]. It has also been reported that apoptotic myoblasts trigger the differentiation of adjacent healthy myoblasts [127]. Thus, a hypothesis has been proposed: conserved biochemical pathways of apoptosis constitute integral components of terminal cell differentiation, and the timing of their engagement and activity levels ultimately determine the cellular choice between death and maturation.

### 4.2. UPS and Autophagy in Skeletal Myogenesis

Skeletal muscle possesses a well-functioning UPS and autophagy system, which effectively eliminate apoptotic nuclear debris and misfolded/toxic proteins, as well as dysfunctional organelles. The proteolytic activities of the proteasome and lysosome may operate in a coordinated manner, whereby degradation of myofibrillar proteins via the proteasome occurs concurrent with the processing of mitochondria and sarcoplasmic reticulum membranes through the autophagy/lysosomal pathway. This coordination could potentially be explained by the existence of common transcription factors, although other possible mechanisms of crosstalk between these two pathways remain to be elucidated. It is precisely the rapid induction of these degradation pathways during myoblast differentiation that facilitates the elimination of pre-existing structures and proteins, thereby driving cellular differentiation and tissue remodeling.

#### 4.2.1. UPS in Skeletal Myogenesis

Previous studies have shown that the UPS acts as both an engine and a brake for cell cycle progression. It controls cell cycle arrest during myogenesis and synergistically regulates the expression of muscle-specific transcription factors to initiate the myogenic pathway. These regulatory effects are specifically dependent on the cell cycle phase and which substrate receptor subunits are assembled into the complex. For instance, the anaphase-promoting complex/cyclosome (APC/C) and Skp1-Cul1-F-box protein complex (SCF) are two major ubiquitin ligases involved in cell cycle control [128]. APC/C, a multi-subunit E3 ubiquitin ligase complex, can activate its ubiquitin ligase activity by directly binding to the adaptor proteins Cdh1 and Cdc20, thereby regulating multiple cell cycle transitions. Cdh1 primarily activates the APC/C from late M phase to the end of G1 phase, and APC/C^Cdh1^ plays a critical role in blocking the G1-to-S phase transition, which is essential for G0 arrest at the onset of differentiation and prolongation of the G1 phase [129]. In contrast, Cdc20 activates the APC/C during G2 and M phases. Substrates of APC/C^Cdc20^, such as securin and cyclin B, undergo ubiquitination and degradation, leading to activation of separase, which triggers sister chromatid separation; inactivation of Cdk1, driving mitotic exit; and completion of chromosome segregation and termination of mitosis [130,131,132]. SCF represents the largest family of E3 ubiquitin ligases, composed of the following four core subunits: Cullin (typically Cullin1), Skp1, F-box protein, and RBX1. These subunits are assembled via the scaffolding role of Cullin1 to form a functional complex. The SCF complex directly binds to substrates through its F-box protein and recruits E2 ubiquitin-conjugating enzymes via RBX1 [133,134]. During the G1-to-S transition and mitotic initiation, SCF ubiquitinates critical cell-cycle regulators (CCRs), including Cyclin E (essential for G1/S progression), Cdc25 phosphatase (activates CDKs to drive cell-cycle progression), and Cip/Kip CDK inhibitors (CKIs) (e.g., p21 and p27, which block CDK activity) [14]. This targeted ubiquitination ensures precise regulation of cell-cycle transitions and mitotic entry.

A growing body of evidence suggests that the UPS regulates myogenesis by targeting muscle-specific proteins. MyoD, a transcription factor that controls cell-cycle exit and muscle-specific gene expression, can undergo ubiquitination at both its N-terminal residues and internal lysine residues. Both types of post-translational modifications appear to direct MyoD for proteasomal degradation [15]. The SCF^MAFbx^ complex has been demonstrated to mediate MyoD ubiquitination at Lys133, leading to its proteasomal degradation. This process requires the muscle-specific F-box protein Atrogin-1/MAFbx, whose overexpression in proliferating myoblasts can antagonize differentiation through induced MyoD degradation [135]. Additionally, studies have revealed that the E3 ubiquitin ligase HUWE1 can induce ubiquitination-mediated degradation of MyoD at both N-terminal residues and internal lysine sites [136]. Post-translational modifications (PTMs) other than ubiquitination also exert regulatory effects on ubiquitination processes. For instance, hyperphosphorylated MyoD has been recognized as a substrate for rapid degradation via the UPS pathway. Nuclear phosphorylation of MyoD at Ser200 triggers its Cdc34-dependent ubiquitination, thereby targeting MyoD for destruction through the 26S proteasome [137,138]. Activated mitogen-activated protein kinase kinase 1 (MEK1) differentiating myocytes phosphorylates MyoD at Tyr156, potentially disrupting the Atrogin-1/MAFbx-MyoD interaction and consequently enhancing MyoD stability [139]. The methyltransferase G9a promotes CUL4-DDB1-DCAF1-mediated ubiquitination of MyoD through methylation, leading to transcriptional repression. This inhibitory effect on myogenic differentiation can be antagonized by the demethylase Jmjd2C [140].

Beyond MyoD, Myog is also regulated by the UPS. For instance, Myog undergoes SCF-dependent ubiquitination and subsequent rapid proteasomal degradation [16]. The von Hippel–Lindau (VHL) tumor suppressor, functioning as the substrate-recognition component of Cullin2-based E3 ligase complexes, mediates VHL-Myog interaction-driven ubiquitination and UPS-dependent degradation [141,142]. Conversely, multiple anti-ubiquitination factors contribute to stabilizing Myog to promote myogenesis. It has been confirmed that TATA-binding protein-interacting protein 120B (TIP120B) enhances C2C12 myogenesis by binding to Cullin1 to inhibit SCF-mediated Myog ubiquitination and degradation [16]. Furthermore, both EGLN3 prolyl hydroxylase and protein 4.1R stabilize Myog, partially through antagonizing VHL-mediated Myog ubiquitination [141,143]. During the early differentiation phase of myoblasts, Myog undergoes USP7-mediated deubiquitination, facilitating its accumulation in differentiating cells to drive terminal myogenic differentiation [144]. At later stages, muscle-specific proteins including titin, myosin heavy chains (MyHCs) and myosin light chains (MLCs) are ubiquitinated by MuRF1 (TRIM63) for proteasomal degradation [145,146]. Collectively, these findings demonstrate the UPS plays stage-specific regulatory roles throughout myogenic progression.

#### 4.2.2. Autophagy in Skeletal Myogenesis

Autophagy in skeletal muscle primarily functions as a non-selective degradation pathway, predominantly associated with macroautophagy, while minor contributions from chaperone-mediated autophagy (CMA) and microautophagy have been proposed. However, CMA and microautophagy have not been experimentally validated in skeletal muscle to date. Distinct from most tissues exhibiting transient autophagy activation during fasting, skeletal muscle demonstrates sustained autophagosome biogenesis persisting for several days [147].

Autophagy is upregulated and functionally indispensable during myoblast differentiation. Inhibition of lysosomal degradation via chloroquine treatment results in marked accumulation of autophagy regulators (e.g., LC3B-II) coupled with reduced p62 levels in differentiating myoblasts, indicating enhanced autophagic flux during this process. Furthermore, pharmacological suppression of autophagy using 3-methyladenine (3MA) (which diminishes LC3B-II) or genetic inhibition via Atg7-targeting shRNA (shAtg7) leads to significant reductions in MyHC protein expression and impaired myotube formation during differentiation [9]. Autophagy in myoblasts is regulated by several conserved signaling cascades [148]. For instance, FoxO3 regulates the transcription of autophagy-related genes including LC3 and Bnip3, a process controlled by phosphorylation-mediated inhibition and activation through upstream AKT and AMPK signaling, respectively. Specifically, Akt phosphorylates FoxO3a at Thr32 and Ser253 residues, leading to its cytoplasmic sequestration via 14-3-3 binding and subsequent functional inhibition. Conversely, AMPK induces activating phosphorylation of FoxO3a at Ser413/588 residues [149,150]. Furthermore, AMPK promotes myoblast autophagy through the following dual mechanisms: direct activation of the ULK1 complex and indirect facilitation via mTORC1 inhibition, which permits ULK1-mediated autophagosome nucleation [151,152].

It is well-known that myoblasts primarily rely on glycolysis to meet their metabolic demands and possess a sparse mitochondrial network, while their differentiation into myotubes—which become highly dependent on oxidative phosphorylation (OXPHOS)—requires significant remodeling of the mitochondrial network. This remodeling involves both mitophagy and biogenesis [153,154,155]. Current research has revealed that during myoblast differentiation, the process is not simply a matter of generating new mitochondria and adding oxidative phosphorylation components. Instead, it involves selective mitophagy to first clear pre-existing mitochondria adapted to glycolysis, followed by the generation of mitochondria with a new metabolic profile. For example, autophagy is strongly upregulated during the early differentiation of C2C12 myoblasts, accompanied by dynamin-1-like protein (DNM1L/DRP1)-mediated fragmentation of the mitochondrial network and sequestosome-1 (SQSTM1)-mediated mitophagy [156]. As differentiation progresses, peroxisome proliferator-activated receptor gamma coactivator 1-alpha (PPARGC1A/PGC-1α)-driven biogenesis replenishes the mitochondrial pool, while the mitochondrial fusion protein OPA1 is rapidly upregulated, leading to the reformation of the mitochondrial network [156,157]. Autophagy inhibitor bafilomycin A1-treated differentiating myoblasts exhibit altered levels of PPARGC1A, DNM1L, and OPA1, along with impaired mitochondrial network remodeling, suggesting that induced mitophagy appears to be essential for mitochondrial network reconstruction and myotube formation. Studies indicate that differentiating C2C12 cells can trigger mitophagy through specific activation of the PTEN-induced putative kinase 1 (PINK1) and Parkin ubiquitin ligase. Notably, knockdown of PINK1 leads to a decline in Myog mRNA levels in differentiating C2C12 cells [157].

Autophagy deficiency during myoblast differentiation exacerbates apoptotic signaling. For instance, 3-MA treatment during differentiation elevates apoptotic rates in myoblasts, and shRNA-mediated ATG7 knockdown (shAtg7) demonstrates increased mitochondrial release of CYCS and AIFM1, accompanied by enhanced activation of caspase-9 and caspase-3 [158]. Analogously, CRISPR-Cas9-generated Bnip3 knockout (Bnip3−/−) myoblasts exhibit significantly higher caspase-9 activity during differentiation, with concomitant increases in caspase-3 activation and DNA fragmentation. Notably, although caspase-3 activation is physiologically required during early differentiation stages, autophagy-deficiency-induced hyperactivation of mitochondrial apoptosis ultimately impairs myogenic progression [158]. Additionally, administration of the chemical inhibitor of caspase-9 (Ac-LEHD-CHO) or dominant-negative caspase-9 (ad-DNCASP9) partially restored myogenesis in shAtg7 myoblasts. This indicates that autophagy modulates mitochondrial-mediated apoptotic signaling during myoblast differentiation and protects differentiating myoblasts from apoptosis [158]. There is also a bidirectional regulatory relationship between apoptosis and autophagy. For example, the Beclin-1/PIK3C3 complex mediates phagophore nucleation, while the binding of Bcl-2 to Beclin-1 inhibits autophagy initiation. Additionally, c-Jun N-terminal kinase 1 (JNK1) and p38 mitogen-activated protein kinase (p38 MAPK) phosphorylate Bcl-2 to promote its dissociation from Beclin-1 and trigger autophagy [159,160,161]. In summary, during myoblast differentiation, the increase in apoptosis activates cellular autophagy, which in turn inhibits apoptosis, preventing excessive cell death and maintaining cellular stability. The bidirectional feedback regulation between apoptosis and autophagy is essential for the differentiation and development of skeletal muscle cells. This regulatory mechanism determines the balance between cell death and survival, enabling skeletal muscle cells to undergo normal differentiation.

### 4.3. Connection Between UPS and Autophagy in Skeletal Myogenesis

A growing body of research highlights crosstalk between autophagy and the UPS in skeletal muscle. Using the same Mlcf-Cre promoter, skeletal-muscle-specific knockout of Atg7 in mice results in upregulation of MuRF1 and Atrogin-1/MAFbx [162]. Conversely, conditional deletion of a proteasome regulatory subunit Rpt3 in skeletal muscle leads to elevated levels of Beclin-1, Atg5, p62/SQSTM1, and LC3-I [163]. These findings collectively indicate that UPS and autophagy are functionally coupled and can compensate for each other to maintain muscle homeostasis. Furthermore, deletion of Rpt3 results in a significant reduction in LC3II conversion (LC3II:LC3I ratio), and the progression of autophagolysosome formation appears to be impaired, likely mediated by an unknown but proteasome activity-dependent mechanism. Interestingly, although both genetic models (Atg7 and Rpt3 knockout) lead to severe myogenic defects, the Rpt3-deficient mice exhibit more pronounced phenotypes compared to controls, including premature death, suggesting that the UPS pathway exerts a greater influence on maintaining skeletal muscle homeostasis [163].

In recent years, nodal molecules connecting autophagy and the UPS have been progressively identified. These molecules participate directly and indirectly in proteolysis to provide resources for maintaining skeletal muscle integrity. For instance, the transcription factor FoxO3 represents a critical link between autophagy and the proteasome system, as it can stimulate the transcription of genes essential for both UPS (Atrogin-1/MAFbx and MuRF1) and autophagy (ULK1/2, Beclin-1, PIK3C3, LC3, etc.). FoxO3 is activated by AMPK and inhibited via phosphorylation by Akt. Additionally, NF-κB participates in stimulating the expression of MuRF1 and Beclin-1, thereby cross-regulating UPS and autophagy [164,165,166,167]. It appears that the major cellular protein degradation pathways heavily rely on ubiquitination, which may serve as a common signal for targeting proteins, protein aggregates, and subcellular compartments to the proteasome or autophagolysosome. The ubiquitin ligase MDM2 suppresses FoxO3 and p53 via the UPS, with the latter also acting as a transcription factor for autophagy-promoting genes [168,169,170]. TNFR-associated factor 6 (TRAF6), possessing E3 ubiquitin ligase activity, catalyzes Lys63-linked polyubiquitination of target proteins, marking them for selective autophagy. Evidence shows that muscle-specific TRAF6 knockout mice exhibit impaired synthesis of proper lysosomal and proteasomal components [171]. The UPS is essential for initiating autophagy; in skeletal muscle, E3 ligases Mul1 and Parkin ubiquitinate outer mitochondrial membrane (OMM) proteins—primarily mitofusin 2 (Mfn2)—which are subsequently recognized by p62 to trigger mitophagy. Nevertheless, the physiological relevance of the proteasome and autophagy in skeletal muscle development remains largely unknown.

## 5. Conclusions

The regulation of myogenic cell apoptosis and autophagy during mammalian skeletal myogenesis represents a tightly coordinated interplay essential for muscle development, regeneration, and homeostasis. Apoptosis ensures the removal of excess or defective myoblasts, refining the cellular pool for proper myofiber formation, while autophagy acts as a critical quality-control mechanism, clearing damaged organelles and misfolded proteins to maintain cellular energy balance and proteostasis. Key molecular regulators, including FoxO transcription factors, AMPK, Akt, and NF-κB, orchestrate these processes by integrating nutrient-sensing, stress-response, and growth factor signaling pathways. Notably, the crosstalk between the UPS and autophagic machinery highlights their synergistic roles in skeletal muscle remodeling, with E3 ligases such as Mul1 and Parkin bridging mitochondrial integrity to mitophagy. Despite these advances, the physiological relevance of dynamic shifts in proteasomal and autophagic activity during distinct stages of myogenesis remains underexplored. Future studies should address how spatial–temporal modulation of apoptosis and autophagy influences satellite cell activation, myonuclear positioning, and muscle adaptation, offering novel therapeutic insights for neuromuscular disorders and age-related sarcopenia.

The intricate connection among apoptosis, UPS, and autophagy during skeletal myogenesis underscores their collective role in maintaining cellular homeostasis and ensuring precise muscle development. Apoptosis eliminates superfluous or dysfunctional myogenic cells to sculpt functional myofibers, while the UPS and autophagy synergistically regulate protein quality control—the UPS rapidly degrades short-lived or misfolded proteins, and autophagy clears bulky aggregates, damaged organelles (e.g., mitochondria via mitophagy), and long-lived proteins. Molecular crosstalk between these pathways is evident: FoxO transcription factors, activated by AMPK or inhibited by Akt, transcriptionally coordinate both UPS components (e.g., Atrogin-1/MuRF1) and autophagic machinery (e.g., LC3 and Beclin-1). Furthermore, E3 ubiquitin ligases such as Mul1 and Parkin exemplify this integration by tagging mitochondrial proteins (e.g., Mfn2) for proteasomal degradation or autophagic clearance, linking organelle dynamics to muscle remodeling. Notably, stress-responsive regulators like NF-κB and TRAF6 modulate both proteolytic systems, highlighting their adaptability in balancing anabolism and catabolism. Despite progress, the temporal–spatial regulation of these pathways during myoblast differentiation, fusion, and maturation remains poorly defined. Unraveling how dysregulation of apoptosis–UPS–autophagy networks contributes to muscle wasting or regenerative failure could unveil therapeutic strategies for neuromuscular diseases and aging-associated sarcopenia. Future studies should prioritize mapping context-dependent signaling nodes and exploring pharmacological or genetic interventions to restore proteostatic equilibrium in compromised skeletal muscle.

## Data Availability

No new data were created or analyzed in this study.
